# Creating an index to measure health state of depressed patients in automated healthcare databases: the methodology

**DOI:** 10.1080/20016689.2017.1372025

**Published:** 2017-09-13

**Authors:** Clément François, Adrian Tanasescu, François-Xavier Lamy, Nicolas Despiegel, Bruno Falissard, Ylana Chalem, Christophe Lançon, Pierre-Michel Llorca, Delphine Saragoussi, Patrice Verpillat, Alan G. Wade, Djamel A. Zighed

**Affiliations:** ^a^ HEOR department, Lundbeck, Deerfield, USA; ^b^ Rithme Consulting, Villeurbanne, France; ^c^ Global Epidemiology Department, Lundbeck SAS, Issy-les-Moulineaux, France; ^d^ Mapi, Nanterre, France; ^e^ CESP, INSERM U1018, Universitté Paris-Sud, Université Paris-Saclay, UVSQ, Paris, France; ^f^ Econometrics Department, Lundbeck SAS, Issy-les-Moulineaux, France; ^g^ Psychiatry Department, Marseille University Hospital, Marseille, France; ^h^ CMP B, CHU Clermont Ferrand, EA Clermont Auvergne, France; ^i^ CPS Research, Glasgow, UK; ^j^ ERIC Lab, Lumière Lyon 2 University, Lyon, France

**Keywords:** Database, depression, health state, index, outcome, cohort

## Abstract

**Background and objective**: Automated healthcare databases (AHDB) are an important data source for real life drug and healthcare use. In the filed of depression, lack of detailed clinical data requires the use of binary proxies with important limitations. The study objective was to create a Depressive Health State Index (DHSI) as a continuous health state measure for depressed patients using available data in an AHDB.

**Methods:** The study was based on historical cohort design using the UK Clinical Practice Research Datalink (CPRD). Depressive episodes (depression diagnosis with an antidepressant prescription) were used to create the DHSI through 6 successive steps: (1) Defining study design; (2) Identifying constituent parameters; (3) Assigning relative weights to the parameters; (4) Ranking based on the presence of parameters; (5) Standardizing the rank of the DHSI; (6) Developing a regression model to derive the DHSI in any other sample.

**Results**: The DHSI ranged from 0 (worst) to 100 (best health state) comprising 29 parameters. The proportion of depressive episodes with a remission proxy increased with DHSI quartiles.

**Conclusion**: A continuous outcome for depressed patients treated by antidepressants was created in an AHDB using several different variables and allowed more granularity than currently used proxies.

## Introduction

The evaluation of pharmaceutical products or medicinal interventions is an essential part of health-related studies. Due to their carefully selected populations, the results of randomized clinical trials of therapeutic interventions cannot necessarily be fully extrapolated to real life [1]. Non-interventional studies, while less precise in defining intrinsic efficacy and safety, provide a much more generalizable knowledge of the performance of the drug or intervention post marketing. Thus both approaches are required to establish healthcare policies and to target the most appropriate intervention for each individual patient [2].

The analysis of data recorded in automated healthcare databases (AHDB), like medical and pharmacy claim records, is of great interest as it provides automatically collected observational data with *a priori* no ‘study participation’ effect [3]. Nonetheless, the automation of the recording process itself limits the collection of additional data from either the patient or the physician. This restricts the ability to confirm the accuracy of the information contained within the database or to derive additional information. In response to this limitation, researchers most often use proxies, which have progressively been developed and are now largely accepted and recommended [4]. For instance, adherence to the treatment can be estimated using the medication possession ratio and more or less narrow time windows can be used to evaluate the patient’s treatment patterns (e.g., concomitant treatments, switch, combination, discontinuation).

The major weakness of AHDB resides in the substantial lack of detailed clinical data. In the field of depression, this absence is hampering since the assessment of the disease outcomes or patient’s health state or well-being in such a disorder cannot usually rely on the data present in these databases (e.g., prescription, delivery or reimbursement of a drug, or the presence or absence of a diagnosis code). For a patient diagnosed with depression, treatment cessation in an AHDB with outpatient data could be the consequence of remission but could also indicate hospitalization or non-adherence to treatment. Similarly, treatment resumption could indicate a relapse of symptoms, but could also be an indicator of improved adherence to the treatment. These problems demonstrate the need for a tool that could be used to evaluate outcomes for depressed patients when assessed in an AHDB.

In response to this problem, proxy measures were developed to assess remission of depression. These, however, are of limited use as they rely only on prescription patterns and do not take medical information into account [5,6]. Furthermore, these proxies are based on the hypothesis that the ‘success’ of treatment in depression can be dichotomized into two separate groups: remitters and non-remitters – a dichotomy that does not fully consider the complexity of this disease [7].

Proxies based on a single or a limited set of variables have significant limitations and so we hypothesized that the incorporation of multiple facets of the data available within an AHDB might produce a composite score reflecting the health state of individual depressed patients treated with an antidepressant (AD), at a specific point in time. Our aim was therefore to build a Depression Health State Index (DHSI) designed to range on a continuous scale from ‘worst possible’ to ‘best possible’ health state. This tool should permit the comparison of patient groups including those defined by different medical treatments or interventions.

This paper describes the development of the DHSI using data from the UK Clinical Practice Research Datalink (CPRD). We describe the process of parameter development, model fitting and testing on an independent sample. Final results and validation will be presented in a separate paper.

## Methodology of the DHSI

### Conceptualization of the index

The aim of this study was to produce an index using as much of the relevant information available in an AHDB as possible. The health state of the patient would be described as a continuous scale from 0 to 100, 0 being the worst possible state and 100 the best possible state. A diverse panel of clinical and methodological experts were constantly engaged for the development of this metric.

The following steps were undertaken:

1. Defining the study design and analytic approach to achieve the objective;
2. Working with clinical and methodological experts to identify potential variables in the database and to derive relevant parameters to estimate the health state of a depressed patient;
3. Working with the experts to assign relative weights to each of the parameters according to their presumed negative or positive impact on the health state, and derive a positive pre-score and a negative pre-score;
4. Sampling and ranking individual depressive episodes according to the score derived from the presence and absence of weighted parameters for each depressive episode;
5. Creating the DHSI by standardizing from 0 to 100 the rank derived in step (4) (i.e., the episode with the lowest rank having a score of 0 and the episode with the highest rank having a score of 100);
6. Using the standardized score in step (5) (i.e., the original DHSI) to develop a regression model and directly derive the DHSI in any other sample using the models’ covariates; and
7. Testing the regression model on an independent sample.

These steps are detailed below.

### Database

The study was performed using data from the CPRD, a database of anonymized primary care records for patients registered at general practices in the UK. It covers approximately 8% of the UK population and includes information on prescriptions of medicines, referrals to hospitals or specialists, and diagnoses entered by the general practitioner (GP) using Read or Oxford Medical Information System codes. This database has been validated for, and is widely used in, pharmacoepidemiological studies [8–10].

### Step 1: study design

The study was based on a historical cohort design using data from the CPRD. The study population comprised patients with at least one depressive episode during the study period (1 January 2006–31 December 2012). The patients were selected based on the following inclusion criteria:

- incident prescription of AD in monotherapy during study period (index date),
- no AD prescription in the 6 months before index date,
- incident diagnosis of depression during the 61 days preceding or following index date,
- patient aged 18 or older at index date,
- at least 6 months of available data before index date,
- at least 9 months of available data after index date (except for patients with a recorded death during this period of time).

Exclusion criteria were lifetime diagnoses of bipolar disorder or of schizophrenia.

**Figure 1.**
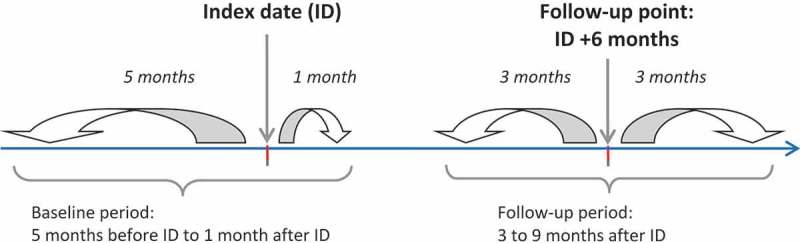
Study design. The index date was the date of the first prescription of antidepressant for a patient meeting the inclusion and exclusion criteria in the database.

For a single patient, these selection criteria could be identified several times during the study period. Each potential time section matching the selection criteria was defined as a ‘depressive episode’: a single patient could have several depressive episodes in the study. The DHSI and its constitutive parameters were described for each individual depressive episode (Figure 1).

Preliminary discussions with the experts lead to consider measuring the DHSI 6 months after index date, a timespan usually considered for assessing depression remission in clinical practice [11]. To achieve this, baseline characteristics for each episode were assessed through events occurring in the 5 months before and 1 month after index date (baseline period). Follow-up characteristics used in the measurement of relevant parameters and computation of the index value were based on events occurring between 3 and 9 months after index date (follow-up period).

### Step 2: identification and definition of the parameters

Selection and definition of the parameters of the DHSI were performed by a group of four clinical and methodological experts including two psychiatrists, one epidemiologist and one UK physician. Experimented analysts of the CPRD presented them with the content of the database (i.e., available data). Then, based on practical and/or theoretical knowledge of the disease, the experts selected variables related to depression directly available in the database and defined derived parameters from these variables thought to impact on the health state of the depressed patient treated with an antidepressant. Parameters were either existing variables in the database or derived from existing variables. The parameters included in the index (Tables 1 and 2) were defined as binary variables (present or absent) and could be incident (e.g., death of the patient) or relative to the baseline period (e.g., dose decrease). Selection and definition of parameters was performed during four multidisciplinary meetings, through discussion and consensus. The current knowledge available and the different ways of assessing depression and treatments in databases, particularly in the CPRD, were considered.

**Table 1.** Parameters with a positive weight in the DHSI.

Var.	Parameter	Definition	Inter-class relative weight	Intra-class value code	Coefficient
X1	No antidepressant prescriptions^a,c^	At least 2 consecutive visits without any prescription for an antidepressant during follow-up period and no ulterior psychiatric prescription during follow-up.	High	3	6^5
X2	No psychiatric co-prescriptions^a,c^	At least 2 consecutive visits without any prescription for any psychiatric co-prescription during follow-up period and no ulterior psychiatric prescription during follow-up.	High	3	6^5
X3	Increasing duration between visits to the GP^b^	Duration between visits to the physician during follow-up period is one standard deviation or more above the duration observed during observation period.	Medium	3	6^4
X4	Decreasing N of other psychiatric co-prescription^b^	A lower number of distinct molecules of psychiatric drugs (other than hypnotics) during follow-up period when compared to reference period (no threshold).	Medium	2	6^3
X5	Disappearance of depression diagnoses^a^	At least one depression diagnostic code during follow-up period but none at last visit(s).	Low	2	6^2
X6	Decreasing N of somatic co-morbidities^b^	A lower number of distinct somatic comorbidities during follow-up period when compared to reference period (no threshold).	Low	1	6^1
X7	Decreasing N of hypnotic co-prescription^b^	A lower number of prescriptions of hypnotic drugs during follow-up period when compared to reference period (no threshold).	Low	1	6^1
X8	Decreasing N of somatic co-prescription^b^	A lower number of prescriptions of somatic drugs during follow-up period when compared to reference period (no threshold).	Low	1	6^1
X9	Pregnancy^a^	Single incident pregnancy recorded during the observation period (excluding deliveries and pregnancies leading to voluntary terminations).	Low	1	6^1
X10	Dose decrease of initial treatment^b^	For patients whose AD molecule is not modified between reference and follow-up periods: the mean daily dose of the complete follow-up period is lower than the mean daily dose of the last month of reference period (no threshold).	Low	1	6^1

^a^ Incident parameter.

^b^ Relative parameter.

^c^ Not retained in the improved linear regression.

**Table 2.** Parameters with a negative weight in the DHSI.

Var.	Parameter	Definition	Inter-class relative weight	Intra-class value code	Coefficient
Y1	Death of the patient^a,b^	Single incident recorded death of the patient during follow-up period.	Highest	3	6^10
Y2	Psychiatric hospitalization^a^	Single incident recorded psychiatric hospitalization of the patient during follow-up period.	High	3	6^9
Y3	Suicide attempt^a^	Single incident recorded suicide attempt of the patient during follow-up period.	High	3	6^9
Y4	ECT prescription^a^	Single incident recorded ECT prescription during follow-up period.	High	2	6^8
Y5	Referral to a psychiatrist^a^	Single incident recorded psychiatrist referral or visit to a psychiatrist during follow-up period.	High	2	6^8
Y6	Sick-leave^a,b^	Single incident recorded sick leave prescription during follow-up period.	High	1	6^7
Y7	Switch^a^	The prescription of a different AD prescribed between 31 days before and 183 days after the initial AD has been stopped. The first AD stop can occur before the follow-up period but new prescription must occur during follow-up period.	Medium–High	3	6^6
Y8	Early termination of pregnancy^a,b^	Single incident termination of pregnancy during the follow-up period.	Medium–High	2	6^5
Y9	Increasing N of other psychiatric co-prescriptions^c^	A higher number of distinct molecules of psychiatric drugs (other than hypnotics) during follow-up period when compared to reference period (no threshold).	Medium–High	1	6^4
Y10	Appearance of a new psychiatric comorbidity^a^	Single incident appearance of a psychiatric comorbidity during follow-up period that is not present at reference period.	Medium	2	6^3
Y11	Combination (AD co-prescription)^a^	The prescription of a different AD than the initial AD any time between the first day after index date and no later than 31 days before the initial AD has been stopped. New prescription can occur at any time after index date but the concomitance of treatment must be observed during follow-up period.	Medium	2	6^3
Y12	Augmentation (AP co-prescription)^a^	The prescription of an antipsychotic or lithium that appears any time between the 1st day after index date and no later than 31 days before any AD treatment has been stopped. New prescription can occur at any time after index date but the concomitance of treatment must be observed during follow-up period.	Medium	2	6^3
Y13	Relapse/recurrence type event^a^	Any prescription for any psychiatric treatment during the observation period between 45 and 183 days after previous AD stop. The new prescription must occur during observation period, but the first AD stop can occur before observation period.	Medium	2	6^3
Y14	Decreased duration between visits to the GP^c^	Duration between visits to the physician during follow-up period is one standard deviation or more below the duration observed during observation period.	Medium	1	6^2
Y15	Dose increase of the initial treatment^c^	For patients whose AD molecule is not modified between reference and follow-up periods: the mean daily dose of the complete follow-up period is higher than the mean daily dose of the last month of reference period (no threshold).	Medium–Low	1	6^1
Y16	Increasing N of somatic co-morbidities^c^	A higher number of distinct somatic comorbidities during follow-up period when compared to reference period (no threshold).	Low	1	6^0
Y17	Increasing N of hypnotic co-prescriptions^c^	A higher number of prescriptions of hypnotic drugs during follow-up period when compared to reference period (no threshold).	Low	1	6^0
Y18	Increasing N of somatic co-prescriptions^c^	A higher number of prescriptions of somatic drugs during follow-up period when compared to reference period (no threshold).	Low	1	6^0
Y19	Hospitalization for other causes^a^	Single incident recorded non psychiatric hospitalization of the patient during follow-up period.	Low	1	6^0

^a^ Incident parameter.

^b^ All cause.

^c^ Relative parameter.

ECT: electroconvulsive therapy.

At time of definition, parameters were separated into two groups:

- parameters with a presumed positive impact on the patient’s health state (progression toward improvement of the health state for the considered episode of depression) (Table 1); and
- parameters with a presumed negative impact on the patient’s health state (progression toward worsening of the health state for the considered episode of depression) (Table 2).

A total of 29 parameters were identified, of which 10 were attributed a positive weight and 19 a negative weight.

### Step 3: assigning weights to the parameters

Next, relative weights were assigned to each of the 29 parameters identified. This important step was necessary to enable the aggregation of different parameters with different metrics into a unique index. The principle of this step was based on a hierarchy of two weightings, according to a qualitative process (i.e., ordered classes and intra-class levels):

- Ordered classes (inter-class relative weight) for each of the positive-weight and negative-weight parameters separately (highest, high, medium-high, medium, medium-low and low);
- Intra-class categorization of parameters sharing the same ordered class was performed by attributing an intra-class ‘value code’ (1: low weight, 2: medium weight, 3: high weight).

For each candidate episode a positive pre-score was derived by summing the weighted positive parameters (the weights being the value codes and coefficients) and, similarly, a negative pre-score was calculated by summing the weighted negative parameters. During this process, positive-weight (X_i_) and negative-weight (Y_i_) parameters were ordered by the clinical experts according to their known importance in terms of impact on the health state using the numerical values. For example, ‘Death of a patient’ (Y_1_) was considered to be the highest possible negative parameter for the DHSI. ‘Psychiatric hospitalization’ (Y_2_) and ‘Referral to a psychiatrist’ (Y_5_) were both ordered as second highest negative parameters – with psychiatrist referral receiving a lower intra-class weight than psychiatric hospitalization. Finally, each qualitative weight (intra- and inter-class) was translated into numerical values: these are detailed in Tables 1 and 2.

All the parameter values were binary. For instance, ‘Psychiatric hospitalization’ was given the value 1 when a psychiatric hospitalization was recorded for this patient during the observation period and the value 0 when no psychiatric hospitalization was recorded.

Two numerical values were associated with each parameter so as to preserve the relative inter- and intra-class ordering when parameters were aggregated. The first numerical value was the ‘Value code’ ranging from 1 to 3, which aimed to preserve the intra-class order (1, 2 or 3). The second numerical value was a ‘Coefficient’ which ranged from 6^0 to 6^10 and aimed to preserve the inter-class order. The value of 6 was selected, as it is the maximum number of variables within a single class. Power values were set to preserve both inter- and intra-class order.

Thus, for a given candidate episode, positive pre-scores were calculated as illustrated in the following example.

Considering an episode with: no antidepressant (X1 = 1) or no psychiatric (X2 = 1) co-prescriptions during follow-up period, an increase in duration between visits to the GP (X3 = 1), a decreasing number of other psychiatric co-prescriptions (X4 = 1), the disappearance of depression diagnoses (X5 = 1), a decreasing number of somatic co-morbidities (X6 = 1), a decreasing number of hypnotic co-prescriptions (X7 = 1) and a decreasing number of somatic co-prescriptions (X8 = 1), no pregnancy (X9 = 0) and no dose decrease of initial treatment (X10 = 0), the positive pre-score would be calculated as (refer to Table 1 for numerical values attributed to the positive parameters):

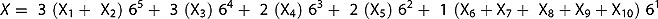

i.e.:

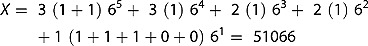

### Step 4: ranking of the episodes of depression

In view of deriving the DHSI, we proceeded by first ranking each depressive episode according to the presence or absence of the parameters defined in the previous step. The ranking step was necessary to obtain, at the end of the process, a unique derived score from the various combinations of parameters

For these steps, feasibility analyses estimated that over 300,000 episodes of depression (and 275,000 individual patients) met the inclusion and exclusion criteria in the available CPRD data.

In view of the development of the regression model (Step 6), a random selection of 90% of the candidate episodes, the ‘learning sample’, was used to develop the DHSI and corresponding regression model. The remaining 10% of depression episodes were left aside as a ‘test sample’ for the validation of the regression model on an independent sample (Step 7).

For each depressive episode of the learning sample, positive and negative ‘pre-scores’ were established according to the presence or absence of positive and negative parameters (Step 3). The episodes were then ranked in ascending order based on the positive pre-score and in descending order based on the negative pre-score. A mean rank of both positive and negative rankings was computed for each individual episode providing the final ranking of the pre-score for all depressive episodes. This ranking step was deemed necessary to merge the information provided by both the negative and positive parameters. As an illustration, four examples of depressive episode ranks (positive and negative pre-ranks and overall mean ranks) are presented in Table 3.

**Table 3.** Illustrative examples of episodes from pre-ranking to normalized DHSI.

	Positive pre-score	Negative pre-score	Positive pre-ranking^a,b^	Negative pre-ranking^a,c^	Overall mean rank^a,d^	HSI^a,e^
Episode 1	50,000	1000	2	4	3.0	80
Episode 2	45,000	15,000	1	1	1.0	0
Episode 3	60,000	2500	4	3	3.5	100
Episode 4	55,000	10,000	3	2	2.5	60

^a^ A higher value indicates a better health state.

^b^ According to ascending order.

^c^ According to descending order.

^d^ (Positive pre-ranking + negative pre-ranking)/2.

^e^ Linear normalization.

### Step 5: normalization of the DHSI

**Figure 2.**
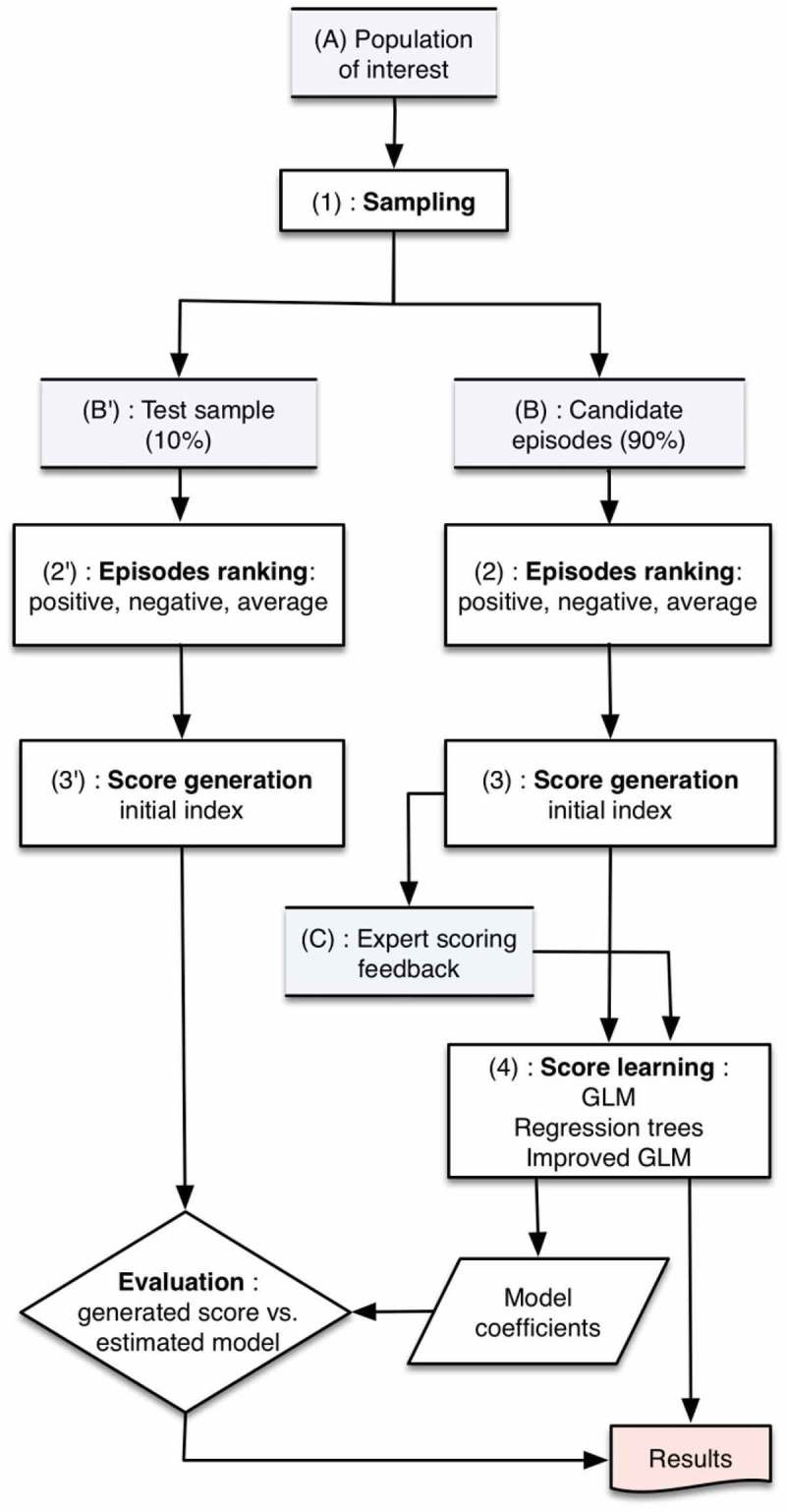
Index creation process.

The previous ranking step (i.e., Step 4) allowed to obtain a unique derived score from the various combinations of parameters. In order to provide a more intuitive and comprehensible metric, this overall rank was then normalized in a linear fashion to a score ranging from 0 (i.e., the episode with the most negative values and the least positive values) to 100 (i.e., the episode with the least negative values and the most positive values) (Table 3). These steps are further detailed in Figure 2.

### Step 6: deriving the DHSI from a regression model

The DHSI was designed to be independent of our specific sample of depression episodes and applicable to any other CPRD extraction. Thus, it was necessary to develop a regression model that could estimate a similar DHSI based on the parameters of the episode without requiring a prior ranking step.

This was performed in two successive steps: (1) creation of a multiple linear regression model (General Linear Modelling algorithm); and (2) improvement of this model using regression trees to identify and take into account potential interactions between covariates (i.e., the parameters).

The initial linear regression was built using the score obtained by the ranking as the dependent variable and all of the 29 (binary) parameters as covariates.

**Figure 3.**
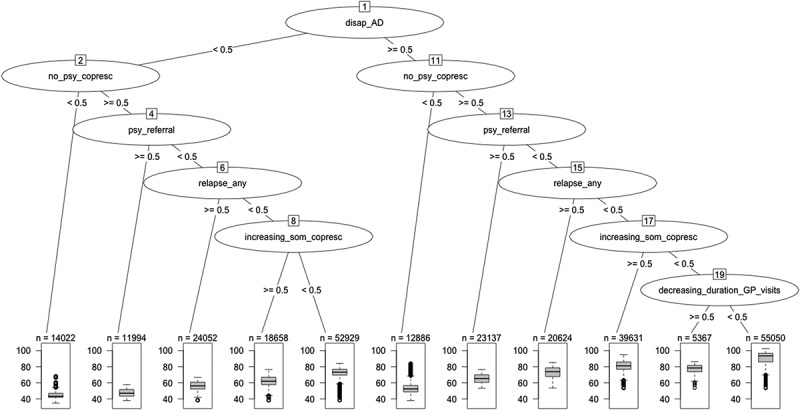
Regression tree to improve the multiple linear regression model.

Improvement of this initial linear regression was performed using a regression tree method, which allowed identifying interactions between the covariates (Figure 3). During this step, the parameter that generated the largest difference in the average score was selected as the most discriminant variable. Then, for each of the resulting two groups of episodes, the same methodology was applied to find the next most discriminant covariate. These steps were repeated until one of the two following stopping criteria was met: size of the terminal node was smaller than 50 episodes; or the overall *R* ^2^ of the model was lower or equal to 0.01 point compared to *R* ^2^ of the model at the preceding step.

A total of 11 potential interactions were identified using this method. These new interaction variables were then added as new variables to the multiple linear regression model. The regression trees also led to the removal of two positive-weight parameters from the linear regression and of one interaction due to collinearity. Accordingly, the improved linear regression included 27 parameters (8 positive-weight and 19 negative-weight parameters) and 10 interaction terms. Statistical fits of the initial and the improved linear regressions are presented in Table 4.

**Table 4.** Statistical fits of the initial and the improved linear regressions.

		Initial model	Improved model
Model fit quality	F-statistic	103,600	129,600
	F statistic *p*-value	<2.2e−16	<2.2e−16
	Multiple *R*^2^	0.9152	0.9451
	Adjusted *R*^2^	0.9152	0.9451
Residuals	Residual standard error (RSE)	6.522	5.248
	Min	−39.938	−44.121
	1Q	−3.979	−2.485
	Median	−0.423	0.203
	3Q	4.342	3.097
	Max	59.063	41.944

The distribution of the scores obtained by running the improved linear regression model was the following: Mean = 58.5, Minimum = 0; Q1 = 49.58; Q2 = 59.36; Q3 = 68.64; Max = 99.99.

By applying this regression model, one should be able to derive the DHSI on any sample of depressive episodes extracted from the CPRD.

### Step 7: testing the regression model on an independent sample

The robustness and generalization capabilities of the model were then evaluated by running it on the test sample. This step consisted in ranking depressive episodes of this sample using the input parameters without any reference to the learning sample. The ranking procedure was identical to the ranking process described above and was normalized from 0 to 100, similarly.

Both the initial regression model and the improved regression model (including interaction terms) were applied on depressive episodes of the test sample to generate the DHSI values for these test episodes. The robustness of these two predictive models was assessed by comparing the normalized ranking score to the DHSI values estimated by each model using the distribution of residual standard errors (Table 5). The distribution of residuals between the ranking score and predicted score on the test sample tended to be less dispersed for the improved regression model compared to the initial regression model, and exhibited a smaller standard error.

**Table 5.** Distribution of residuals from the difference between the score derived from episode ranking and the score predicted by the regression model. This distribution is presented for the initial regression model and the improved regression model (test sample).

	Initial regression model	Improved regression model
Residual SE	5.886	3.970
Mean	−0.152	−0.103
Median	0.037	0.025
IQR	[−3.031; 3.213]	[−2.045; 2.167]
Min; Max	[−35.160; 45.440]	[−23.720; 30.650]

SE: standard error; IQR: interquartile range.

In addition, the comparison of residual errors generated by the improved regression model on the test sample to those generated by the same model on the learning sample showed very close values. This supports robustness of the DHSI estimator.

### Primary comparison of the score

**Figure 4.**
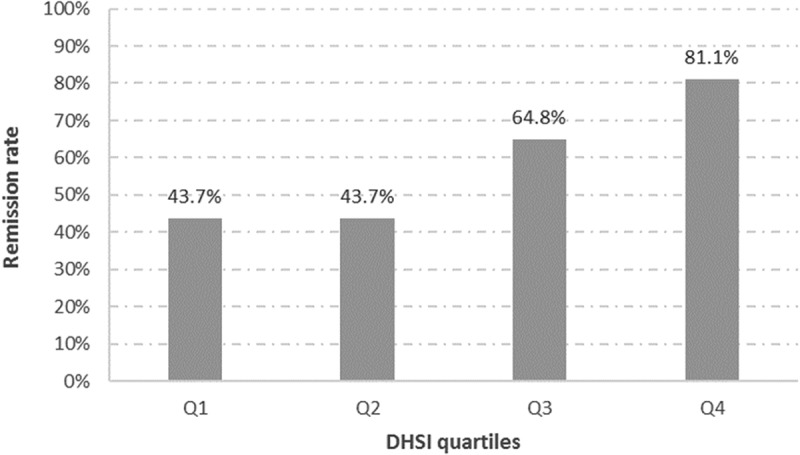
Remission (defined by treatment patterns) rates according to the quartiles of the number of patients for the DHSI.

After completion of the DHSI, we described the score by quartiles (Table 6) and compared them to an existing proxy of remission in database based on treatment patterns of antidepressants [6]. Remission was defined as the absence of antidepressant prescriptions in the database for a period of 45 days or more during the follow-up period of a depression episode. Figure 4 describes the observed remission rates for patients within each quartile of the DHSI. The remission rates tended to increase for each increasing quartile of the DHSI (from 43.7% for the two first quartiles to 64.8% and 81.2% for the last two quartiles).

**Table 6.** Score range of the DHSI according to the quartiles of the number of patients.

Quartiles	N of episodes	% of total episodes	Health State Index (range)
Q1	77,299	25.0%	[0–50]
Q2	71,480	23.1%	[51–60]
Q3	79,305	25.6%	[61–69]
Q4	81,195	26.3%	[70–100]

## Discussion

The overall principle of the DHSI is to aggregate all relevant data contained in an AHDB (here the CPRD) into a unique index reflecting the severity of an episode of depression, from 0 to 100. This tool aims to compensate for the unavoidable lack of detailed clinical data in healthcare databases and provides useful information regarding the health status of an antidepressant-treated patient suffering from an episode of depression.

Currently, the outcome of depression in observational studies of depression using secondary data is frequently analysed using binary outcomes of remission [5,6]. However, depression and its evolution over time are more complex than a binary separation. Whereas patients with a score lower than 10 on the Montgomery–Åsberg Depression Rating Scale (MADRS) are declared remitters, patients with a score ranging from 10 to 60 are declared non-remitters [12]. But it can be argued that patients with a MADRS score of 10 are much closer to patients with a score of 9 than to patients with a score of 40. A tool such as the DHSI could provide further granularity to the description of depressed patients in databases, either during the acute phase of the disease or after a potential ‘treatment success’. Higher granularity will also limit misclassification and favour more precise and sensitive results when comparing evolution of the disease between treatments, time points, disease management strategies or even geographical regions. Indeed, the remission rates tended to increase for each increasing quartile of the DHSI (from 43.7% for the first two quartiles to 64.8% and 81.2% for the last two quartiles) supporting the hypothesis that such an index can provide more granularity than a binary definition. The importance of such a tool is strengthened by the usual lack of scale data in healthcare databases.

With the increasing use of AHDB in pharmacoepidemiology studies, proxies have been developed to respond to this need for higher granularity. A current proxy for the remission status of depression in AHDB stems from the analysis of a computerized Spanish prescription database and the CPRD (ex-GPRD) [6]. However, this definition does not include any clinical data, which is the case for all the current remission proxies used in database analyses, focusing mainly on the presence or absence of an antidepressant prescription. This cannot be fully satisfactory because, like in many other chronic disorders, the absence or presence of a prescription for an antidepressant can reflect either worsening or improvement of the episode of depression. An index based on the aggregation of multiple information should help circumvent this issue related to relying solely on prescription data to define a clinical status (i.e., remission).

A study particularly highlighted the benefit of electronic medical record (EMR) data over claims data [13]. This resulted in the recent development of databases that combine both claims data and EMR data for health care analysis. Indeed, whereas neither claims data nor EMR data alone allow for optimal analysis of patient health status, but rather the best practice of health analysis depends on using both of these data types together, this type of database will address some of the limitations we highlighted in this article (e.g., lack of clinical information). However, we believe that our approach remains relevant as this type of database is still at the early stages. The linkage of EMR and claims data is still limited and not accessible for all existing claims or EMR databases. In addition, the information in the EMR may not be standardised and will differ from one practice to another. Finally, our method and index could be modified to also include information from the claims on top of current information and from the EMR, and will improve the index accuracy.

The creation of the DHSI has proved to be an ambitious goal, in particular, the use and merging of information with different metrics (e.g., binary, continuous…) into a single measure. The index eventually includes a list of 29 parameters, most of which are not based on antidepressant prescription data and represent a substantial proportion of the data related to depressive episodes and available in the CPRD. Parameters were initially identified and selected by clinical and methodological experts with the aim of clinical relevance and methodological consistency. For each parameter, direction and weights on the estimated health state of the patient during a particular episode of depression were provided through multidisciplinary discussions. The development of a regression model allowed the estimation of the index on any sample extracted from the CPRD. The addition of interaction terms identified through regression tree analyses further improved this model.

The methodology used to build this index, discussions with a multidisciplinary expert team, listing and discussion of relevant parameters as well as their weights and rank episodes/patients according to their parameters is easily reproducible. Thus, while the parameters identified in the CPRD may not be available for another AHDB, the global methodology can easily be adapted to any database, and a specific version of the DHSI be created.

Nonetheless, as for any tool, and despite the efforts that were made to ascertain the robustness of this method, some limitations apply. The first limitation is that some important parameters are possibly missing from the list we have identified, because the data itself was missing in the database or because the multidisciplinary group overlooked them. In addition, a misclassification bias leading to over or underestimation of the frequency of the different parameters cannot be excluded. Indeed, the identification of the parameters depends on the Read and British National Formulary code lists used to define these parameters. These code lists used were based on the current knowledge of these classifications at the time of the index construction, and could be updated or improved for future analysis using the DHSI. Another potential limitation is related to the directions and weights attributed to the parameters: these were decided by the working group but may be subject to discussion. Validation of the parameter list, as well as their relative weights, with an extended expert panel could help to determine the extent of these limitations. Sensitivity analyses challenging these relative weights should also be performed to assess the stability of the index.

Limitations linked more specifically to the identification of the population can also be mentioned. The selection criteria included two conditions, which are classically applied in depression studies: the date of an incident AD prescription acting as the index date and a depression diagnosis in a window of 2 months around the index date [14–16]. Nonetheless, while these conditions aim to provide high specificity in the inclusion of the patients, some relevant patients may not have been included in this study (depressive patients not fulfilling one of these criteria). But considering the large number of depressive episodes fulfilling the selection criteria, the risk of including ‘false positives’ (i.e., non depressed patients) by removing one of these criteria would have surpassed the benefit of adding other episodes to this already large sample.

Because the lack of information regarding the health state of the patients recorded in databases often hampers generalizability of the studies conducted using healthcare databases, an additional step of external validation of this index could be useful. This would allow determination of the strength of the index in discriminating the severity of depression and health state of the patients. This could be performed by measuring and comparing the DHSI in populations known for displaying different health states (e.g., according to the region available in the CPRD).

Improvement of the DHSI should also be obtained through its use in different cohorts from the same AHDB, but also through its adaptation to other AHDBs and to other chronic conditions (e.g., other psychiatric conditions). In parallel, this would improve transversal comparisons between AHDBs and disorders, which is one of the major aims of the DHSI. Finally, as the methodology used is easily reproducible, the intrinsic principle of this DHSI can be generalized to other data sources such as data from clinical trials or observational field studies to provide a comprehensive measure of the patient’s health state.

In conclusion, this description of the building of a health state index shows that the apparently complex development of a continuous depression outcome is feasible using easily accessible data. The finalization of this DHSI is ongoing but the results from the initial steps of the creation, and further validation of the DHSI by comparison to classical outcomes, are very encouraging. Next steps include the validation of the parameter choices and weights among other experts and using the index to identify differences between populations to show its epidemiological and clinical relevance. We encourage other research teams to use the open-source publication of this index and its methodology to conduct further researches and refine this index, in depression but also in other conditions.
